# Recruiting older people to a randomised controlled dietary intervention trial - how hard can it be?

**DOI:** 10.1186/1471-2288-10-17

**Published:** 2010-02-22

**Authors:** Sarah E Forster, Laura Jones, John M Saxton, Daniel J Flower, Gemma Foulds, Hilary J Powers, Stuart G Parker, A Graham Pockley, Elizabeth A Williams

**Affiliations:** 1 Department of oncology, Faculty of Medicine and Dentistry and Health, The University of Sheffield, Royal Hallamshire Hospital, Glossop Rd, Sheffield, S10 2JF, UK; 2 Centre for Sport and Exercise Science, Sheffield Hallam University, Collegiate Crescent Campus, Sheffield, S10 2BP, UK; 3 Sheffield Institute for Studies on Ageing, School Faculty of Medicine, 2nd Floor, Samuel Fox House, Northern General Hospital, Herries Road, Sheffield, S5 7AU, UK

## Abstract

**Background:**

The success of a human intervention trial depends upon the ability to recruit eligible volunteers. Many trials fail because of unrealistic recruitment targets and flawed recruitment strategies. In order to predict recruitment rates accurately, researchers need information on the relative success of various recruitment strategies. Few published trials include such information and the number of participants screened or approached is not always cited.

**Methods:**

This paper will describe in detail the recruitment strategies employed to identify older adults for recruitment to a 6-month randomised controlled dietary intervention trial which aimed to explore the relationship between diet and immune function (The FIT study). The number of people approached and recruited, and the reasons for exclusion, will be discussed.

**Results:**

Two hundred and seventeen participants were recruited to the trial. A total of 7,482 letters were sent to potential recruits using names and addresses that had been supplied by local Family (General) Practices. Eight hundred and forty three potential recruits replied to all methods of recruitment (528 from GP letters and 315 from other methods). The eligibility of those who replied was determined using a screening telephone interview, 217 of whom were found to be suitable and agreed to take part in the study.

**Conclusion:**

The study demonstrates the application of multiple recruitment methods to successfully recruit older people to a randomised controlled trial. The most successful recruitment method was by contacting potential recruits by letter on NHS headed note paper using contacts provided from General Practices. Ninety percent of recruitment was achieved using this method. Adequate recruitment is fundamental to the success of a research project, and appropriate strategies must therefore be adopted in order to identify eligible individuals and achieve recruitment targets.

**Trial registration number:**

ISRCTN45031464.

## Background

Some of the difficulties associated with recruiting participants to research studies have been well documented [1,2]. Less than a third of the Medical Research Council (MRC) and the Health Technology Assessment programme-funded studies in the UK that were recruiting between 1994 and 2002 achieved their recruitment targets [1]. Effective strategies to recruit participants should therefore be sought, and the findings shared.

Many reasons have been reported by potential participants for their unwillingness to participate in research, including the demands of the research, the time commitment, treatment preferences, not wanting to give a blood sample and distrust of the research process [3,4]. The principal reasons for agreeing to participant in research are 'considering the research to be important', 'wanting to help researchers' and 'having time' [5]. A US study recently reported that positive media coverage increases 'volunteerism', whereas negative coverage does not appear to adversely affect recruitment [6]. This finding may not of course be readily extrapolated to other societies. Older people provide additional research challenges, and poor recruitment and retention rates are often reported as a consequence [7]. Ill-health, carer responsibilities, suspicions of research or the belief they will not be useful to the researchers can all contribute to poor recruitment in this group [4]. Recruiting elderly people from the community can be particularly time-consuming, often involving large screening samples.

In order to accurately predict recruitment rates, researchers need information on the relative success of various recruitment strategies. Few published trials include such information and often the number of participants screened or approached is not quoted. This can jeopardise the success and feasibility of future studies. The CONSORT guidelines which have been adopted by many journals were devised to improve the quality of reporting in randomised controlled trials. Compliance with the guidelines requires researchers to report details on the participants that were assessed for eligibility, as well as those that were excluded [8]. Nevertheless the methods used to recruit are seldom described.

We have conducted a randomised controlled trial of older people which involved the recruitment of 217 individuals aged 65-85 years from the community to a 6-month randomised controlled dietary intervention. Here we present the recruitment strategies used, to inform other researchers conducting projects either in the community or with older people, as well as funding bodies involved in the financial support of such research.

## Methods

### Overview of the project

The study was designed to examine the relationship between diet/nutrient status and immune function in older adults and to investigate the effect of a dietary intervention on risk of infection and immunological function in older people (Food and Immunity Trial: The FIT study). After recruitment the participants were randomised to one of three treatment arms and received a daily placebo or micronutrient tablet, or were required to incorporate foods rich in certain vitamins and minerals into their diet for 3 months. Subjects were followed for a further 3 months after the intervention and therefore participants were on the trial for 6 months in total. Table 1 outlines the commitment required by the participants during the trial.

**Table 1:** Participant involvement in the FIT study

Involvement required from participants	Associated activity
1 home visit	Orientation to the study, informed written consent, explain food diary ~1 hour

3 hospital visits for assessments: Baseline, post intervention, follow-up	Questionnaires, bloods, anthropometric measures, checking food diary ~1 hour per assessment

1 hospital visit for vaccination	Bloods, vaccination ~30 min

Completion of symptoms and illness diary for 6 months	Weekly ~10 min

Completion of 3 food diaries for 4 days each (recording of food and drink eaten)	Daily ~30 min for 4 days on 3 occasions

Consumption of tablet or specific foods for 3 months (food was paid for by the trial and was delivered to the participants home)	Swallowing of tablet or consumption of provided food over the week for 12 weeks

Eight telephone calls at intervals throughout study	Interviewed re: health and consumption of food (if on food group) ~10 min each call

### Exclusion/inclusion criteria

Participants were included if they were aged 65 to 85 years old, had not taken vitamin and mineral tablets in the last 3 months, had not been hospitalised in the last year and had no severe medical conditions, including those likely to affect their immune system. Potential participants were excluded if they had insulin-dependent diabetes, were unable to comply with the intervention (allergies, dislike of certain foods, difficulties swallowing tablets) or reported consuming 3 or more portions of fruit and vegetables per day. They also were also excluded if they had been given a Tetanus vaccination in the last 5 years, as one of the outcome measures for the study was the influence of the intervention on an individual's immune responsiveness to tetanus vaccination.

### Recruitment

The study was conducted in Barnsley, South Yorkshire, UK which has a population of 218,000 [9,10].

Due to the logistical demands of the immunological analysis of the blood samples and consideration of the frequency of blood collection, a maximum of 3 participants could be recruited each week with a recruitment target of 10 per month.

The recruitment process for the project was divided into 4 parts:-

a) Approach

b) Screening

c) Consent

d) Assessment

Potential participants were identified through eight recruitment strategies and were screened for suitability through discussion either by telephone or in person. Suitable participants were then visited at their home for the purposes of orientating recruits to the study and for the collection of informed written consent. Finally, participants attended the hospital for their first assessment visit and were randomised onto the trial. Following an initial expression of interest, volunteers were informed that on completion of the study they would receive a payment of £100.

This study was approved by Barnsley NHS Ethics Committee (ref: 05/Q2304/48) and was registered as a controlled trial: ISRCTN45031464. All researchers involved had honorary contracts with the Healthcare Provider (The National Health Service, NHS).

## Results

Two hundred and seventeen people were successfully recruited to the trial over a 24-month period (July 2006-July 2008). Seven participants dropped out during the intervention, and one individual dropped-out during the follow-up period.

### Recruitment strategies

#### Recruitment through General Practice

General Practices in the local Primary Care Trust were contacted by a letter sent to the practice manager. The letter introduced the researcher, outlined the study and requested cooperation with the identification of eligible participants from the Practice. The letter was followed by a telephone call in order to provide more information about the study and to discuss requirements of the research. Although the researcher offered to visit the Practice, the majority did not feel this was necessary. Practices were asked to identify all their registered patients aged 65 to 85 years. The majority of Practices chose to give the names and addresses directly to the researchers who then sent letters of invitation and patients information sheets to the potential participants. Invitation letters and information sheets about the study were printed on NHS headed paper and were sent by the researchers to potential participants with a pre-paid envelope. Other Practices preferred to send the letters of invitation and patient information sheet to their eligible patients themselves with stamps provided by the research team. Letters sent from General Practices directly were sent using the Practices own headed paper which included NHS headed logo and also included prepaid envelope. Interested patients were required to return a reply slip and to provide a contact telephone number. Researchers then contacted potential participants by telephone to screen them for eligibility. Whether participants were contacted to by the researchers directly or through the General Practice, the same letter was received by the potential recruit and there was no pre notification for those recruits sent letters directly from the General Practice.

Strategies were devised to enable maximum recruitment for minimum time involvement for the researcher. Size, location and accessibility of the General Practices were considered before they were invited to become involved with the research. Also General Practices in areas of lower socio-economic status were approached first, as they were considered to have more patients with low fruit and vegetable consumption, which was one of the specified inclusion criteria. Twenty General Practices were approached in total, including 4 surgeries with 2 branches. Sixteen Practices agreed to take part. The 4 Practices that declined reported a lack of time for research. The number of patients aged 65-85 years at each Practice varied from under 150 to over 1500.

In total, 7482 letters were sent to potential recruits from the names and addresses supplied by General Practices. Five hundred and twenty eight individuals responded and were screened by telephone. One hundred and ninety five individuals were found to be suitable and consented to take part in the study. Ninety percent of the total participants were recruited by this method. Seven percent of participants approached by letter with addresses supplied by the General Practice responded and were recruited onto the trial.

#### Recruitment through other methods

Seven other methods were also employed to target the 65-85 year age group and increase recruitment.

• Barnsley Metropolitan Borough Council 's Central Call Service, which supplies personal alarms to residents in the Barnsley area provided names and addresses of potential recruits (200 letters sent, 9 responded, 3 recruited).

• Posters and leaflets were given to community groups and 2 advertisements were placed in the local newspaper (Barnsley Chronicle, circulation 40,500) (15 responded, 7 recruited).

• The Post Doctoral Researcher was interviewed about the trial by two local radio stations (BBC Radio Sheffield and Dearne FM) (no response).

• Members of the research team made presentations to a range of groups including the Women's Institute and Friendship groups (4 responded, 4 recruited).

• Arrangements were also made to have a stand in a local supermarket ASDA and market (open 5 times a week, 300 stalls) (6 responded, 1 recruited).

• Participants who were recruited onto the trial were given leaflets and asked to give them to friends and family (15 responded, 7 recruited).

Overall 49 potential recruits responded to recruitment methods other than through the General Practices, and 22 of these started the study. This accounted for 10% of the total participants recruited.

### Screening

**Figure 1:**
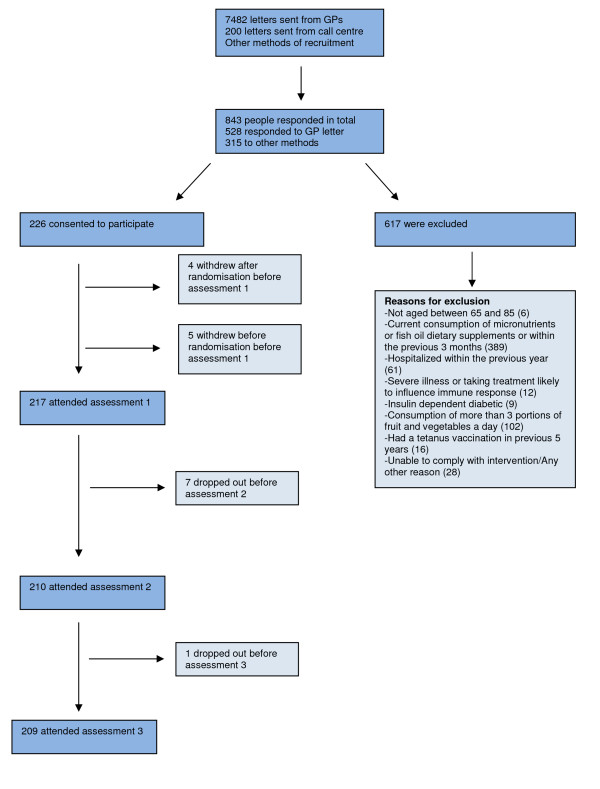
**Number of participants screened and started on the trial including reasons for exclusions**.

When an individual had expressed an interest in the study they were screened for eligibility. The reasons for exclusion are shown in Figure 1. The screening was done by telephone and included a detail description of the study. At this point participants were told of the payment they would receive for taking part. Eleven percent of participants approached directly by letter responded positively requesting information. After the initial telephone screening, 27% of all those who responded were suitable to take part. The main reason for exclusion to this trial was the consumption of micronutrients, which accounted for 63% of exclusions. Of those people found to be suitable, only 3% declined to take part.

### Home visit

All participants were visited at their home by the postdoctoral researcher prior to starting the trial. This gave the researcher another opportunity to explain the study and for the participant to ask further questions. It was also the time at which informed, written consent was taken. Spouses or carers were often present at this visit. This was perceived to be a benefit to the subject and the researcher, as some participants reported difficulties in remembering all the details of the study, despite comprehensive written and oral instructions.

## Discussion

Two hundred and seventeen participants were successfully recruited to the FIT study using a variety of methods over a 24-month period. Writing directly to potential participants from names supplied from General Practices was found to be the most successful recruitment approach. There was no evidence that a letter sent directly from the General Practice yielded a better response than if sent from the researchers. However it should be noted that all communication with participants was on NHS headed paper. Retention of participants to the trial was high, with only 7 subjects dropping out between starting the study and completing the intervention. There was only one additional withdrawal from the study in the follow-up period, thereby resulting in an overall dropout rate of 4%.

### Finding recruits

Writing directly to participants from health authority lists has previously been shown to be successful [11,12]. Menon et al (2008) argued that this method results in a more representative population sample and allows for controlled over-recruitment rates. We also used several other recruitment strategies which contributed 10% of our recruitment target. However, these methods were less predictable in their response rate. The use of multiple methods has been shown to be successful by others [13].

Ethical approval to send reminder letters to participants was not requested and in retrospect this might have helped the recruitment rate after the initial contact letter. Others have shown a follow-up phone call increases recruitment substantially [14], although confidentially issues might mean that ethical permission to do this is not given if names are initially obtained from the General Practice [15].

Although the study only required participants to be enrolled in the trial for 6 months this did involve 1 home visit and 3 hospital assessment visits. Participants randomised to the food group were asked to change their diet and although the food the participants were asked to consume was provided free of charge, this dietary change may have been challenging for some individuals. The demands and challenges of the trial are likely to have influences an individual's decision as to whether to take part in the trial. However, we have no way of knowing if this was the case.

### Inclusion of all suitable participants

Barnes et al. (2005) reported problems with the exclusion of patients due to reasons other than the stated exclusion/inclusion criteria [16], and the use of physicians as trial 'gatekeepers' can reduce recruitment [17]. However, in this study, age was the only inclusion factor that General Practitioners had to consider when identifying eligible individuals registered at their Practice. Care was also taken by the researchers to ensure that only those individuals not fulfilling the inclusion criteria were actually excluded. The researchers strived throughout to include all eligible individuals regardless of the logistical challenges that they presented, such as not having a telephone, inability to speak on the phone, concentration problems and mobility difficulties.

Other researchers have reported that preference to a specific treatment arm can result in poor recruitment to intervention trials [18], therefore problems relating to a participant's preference to a particular treatment arm were anticipated. A third of the participants were randomised to receive approximately £15 worth of food each week, paid for by the study and delivered to their home. The potential provision of free food may have acted as a recruitment incentive for some participants and then subsequently resulted in drop-out if they were not randomised to that treatment arm. Four participants withdrew after randomisation, but prior to being told of their treatment allocation. There was therefore no suggestion that treatment preference caused withdrawal from this trial. The researcher was careful to clearly explain to all participants from the outset that only a third of participants would receive food and that the allocation to treatment was randomised. The recruiting researcher also observed that some participants preferred the micronutrient/placebo tablets, as they were easier to take.

### Maintaining recruitment rates

Anticipating recruitment challenges and calculating projected recruitment rates based on those already recruited is important for deciding whether new recruitment strategies are necessary once the process has begun. Documenting and recording the number of participants that have been screened, and the reasons for exclusion can help with the future planning of recruitment. Others have reported that slower than anticipated recruitment can be compensated for by small numbers of dropouts [5,13], and our experience is similar. However, care must be taken not to rely on this method, as other uncontrollable factors might influence dropouts and anticipated dropout rates must be evidence-based.

### Retaining participants

Minimising respondent burden is likely to maximise response rates at the recruitment stage of a trial [19,20]. Potential recruits initially needed only to complete a reply slip, researchers contacted them subsequently by telephone, to gain further eligibility details.

The relationship between the researcher and the participant has been shown to be important for retaining study participants [21,22]. Our experience is consistent with this and we had very good retention to the trial. Consideration of individual participant's needs and the removal of any obstacles to completing the research can help to ensure high rates of retention. Older people can have difficulties with hearing and vision and associated difficulties with recruiting older people have been noted previously[23].

Older people have been shown to have difficulties with understanding healthcare information due to poor literacy skills [24] and the self completion of the questionnaires required literacy skills. Strategies aimed at helping participants with reading and writing difficulties such as getting help from partners and relatives were therefore adopted. When necessary, the researcher read the questions aloud to the participant and entered correct answers on the questionnaire. Extra time had to be planned to allow for this to be done. Encouragement and reassurance were also very important in giving the participants confidence to complete the task.

Financial incentives have been shown to increase compliance to trials [25]. In this trial, all participants were compensated for their time with £100 on completion of the study, although potential participants were not told of the amount of compensation until they had responded to an initial letter. It is therefore unlikely that financial reward influenced recruitment, however it might have helped retention.

### Strengths and limitations of the study

Older people provide unique challenges for researchers. Although the expansion in ageing research means that a better understanding of the particular challenges that are associated with the recruitment of older people is paramount, few papers have systematically described such challenges and their solutions. Information about recruitment strategies for randomised controlled trials will therefore assist and inform future researchers and their financial supporters.

It must be noted that all participants in this study were white Caucasian, and it is possible that additional issues affect participants from different ethnicities. Participants were recruited from a community setting and although some of the issues will be similar in an acute care setting, some differences may be observed. Considering the area from which recruitment took place, it is likely that our participants were of low socio-economic status. The financial incentive might therefore have had a greater influence on retention to the trial than it would have had were the trial to have been conducted in a more affluent area. Participants were not asked to explain their reasons for taking part in the study, and it was not possible to obtain information from those who did not respond. Table 2 provide key messages for recruitment of older adults to trials.

**Table 2:** Key messages for recruitment of older people to trials

Set realistic recruitment targets
Include time for setting up recruitment initiatives

Include time for screening

Include time for known periods when recruitment might be lower (holidays, seasonal differences, annual leave)

Reassess recruitment strategy once the study as started and be prepared to change accordingly

Be aware of individual participant needs

Where possible use the same researcher to assess the participant throughout the study

Where ever possible include all suitable recruits/avoid recruitment bias

## Conclusion

It is difficult to give a precise and definitive 'instruction manual' on how to find recruits, as inclusion/exclusion criteria to the study as well as the population being recruited will dictate the best methods. Adequate recruitment is fundamental to the success of a research project and appropriate strategies must be adopted to obtain the recruits. This paper gives detailed information of recruitment to a randomised controlled trial of older people, which can be used to help inform others. In our experience the most successful recruitment route for recruitment to a 6 month dietary intervention trial in older adults was found to be by writing directly to potential recruits having identified them via local General Practices.

## Key points

• Writing directly to participants using names and addresses provided by General Practitioners provides the easiest way of recruiting a large number of participants to a trial of older adults.

• Considering the needs of older people is vital to successful recruitment and retention to trials.

• Appropriate time must be allocated to the screening and recruitment process.

## Competing interests

The authors declare that they have no competing interests.

## Authors' contributions

SF and LJ were involved in design of recruitment strategies, recruitment of participants and writing of the manuscript. JS, GP, SP and HJP were involved in design of study and commented on manuscript. DF and GF analysed blood samples. EW was Principal Investigator and was involved in the design of study and recruitment strategies, and in the writing of manuscript. All authors have read and approved the final manuscript.

## Pre-publication history

The pre-publication history for this paper can be accessed here:

http://www.biomedcentral.com/1471-2288/10/17/prepub
